# Subcutaneous Emphysema and Pneumomediastinum after Inhaling Pepper Spray

**DOI:** 10.5334/jbr-btr.988

**Published:** 2016-01-28

**Authors:** Sofie Woussen, Marc Lemmerling, André Verstraeten

**Affiliations:** 1 AZ Sint-Lucas, Ghent, BE

**Keywords:** Pneumomediastinum, Pepper spray

A 25-year-old police officer presented to the emergency department with shortness of breath developed after multiple exposures to pepper spray during training exercises. Physical examination revealed crepitus on palpation and auscultation in the neck, face and thorax, consistent with subcutaneous emphysema. The patient was hemodynamically stable. Blood tests showed no abnormalities. The anteroposterior (AP) chest radiograph (Fig. A) demonstrated diffuse subcutaneous emphysema (solid white arrow), a small pneumothorax in both apical lung fields (solid black arrowheads) and air outlining the thoracic aorta (hollow white arrow), the bronchial branches (hollow black arrowhead), both sides of the diaphragm (hollow black arrow) and both kidneys (solid black arrow). The lateral chest radiograph (Fig. B) showed a well-defined lucency surrounding the right pulmonary artery, also known as the ring around the artery sign (solid black arrow). These radiographic findings suggested the presence of a pneumomediastinum and pneumo(retro)peritoneum. A CT of the chest (Fig. C) confirmed the diagnosis of pneumomediastinum (hollow black arrowhead, hollow black arrow and hollow white arrow) associated with diffuse subcutaneous emphysema (solid white arrow), pneumo(retro)peritoneum (solid black arrow) and pneumothorax (solid black arrowhead). The patient was hospitalized and treated conservatively with rest and oxygen therapy. Subsequent chest radiographs showed a resolution of the mediastinal and intra-abdominal air.

**Figure A:**
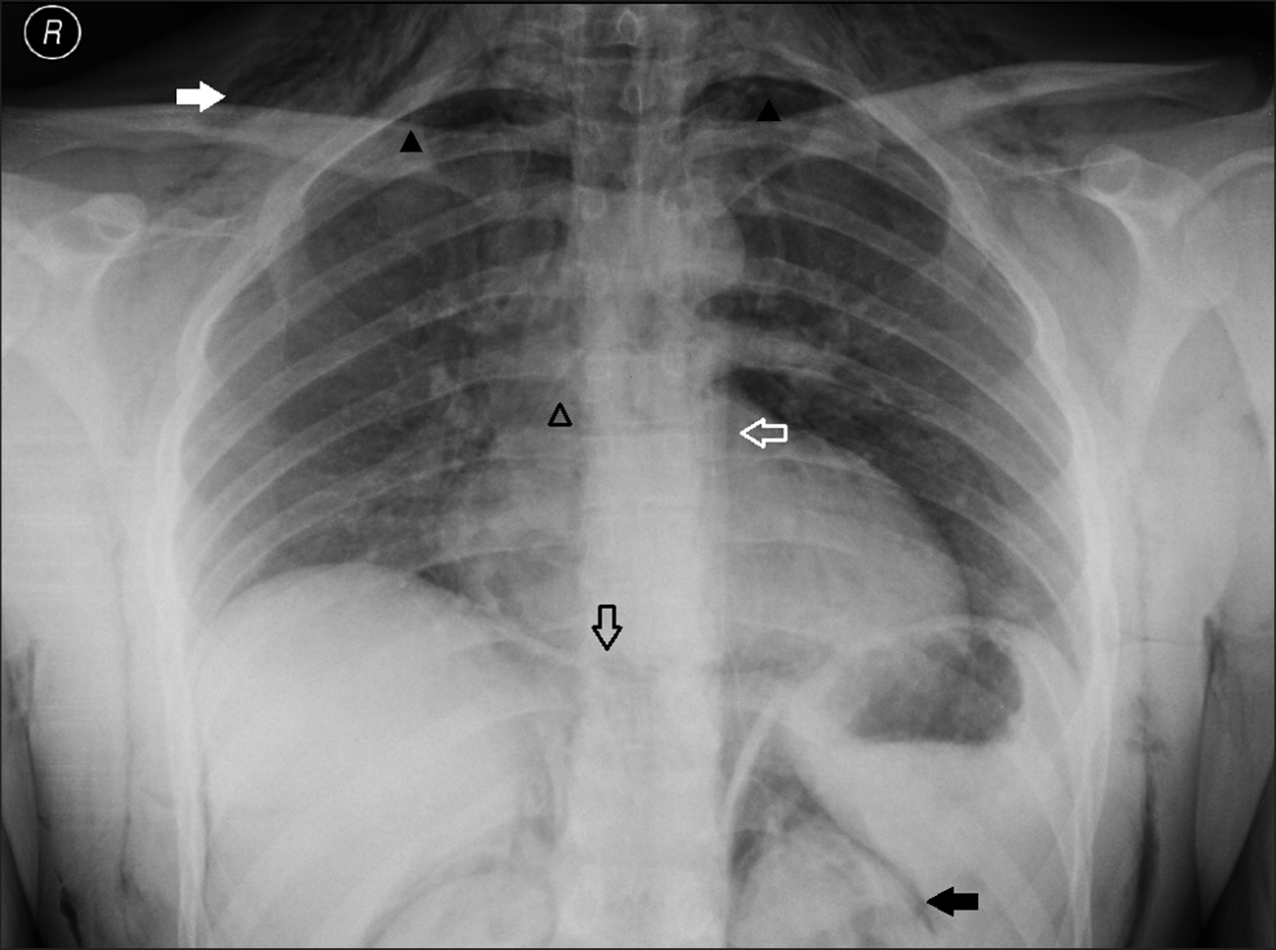
Anteroposterior (AP) chest radiograph demonstrating diffuse subcutaneous emphysema (solid white arrow), a small pneumothorax in both apical lung fields (solid black arrowheads) and air outlining the thoracic aorta (hollow white arrow), the bronchial branches (hollow black arrowhead), both sides of the diaphragm (hollow black arrow) and both kidneys (solid black arrow).

**Figure B:**
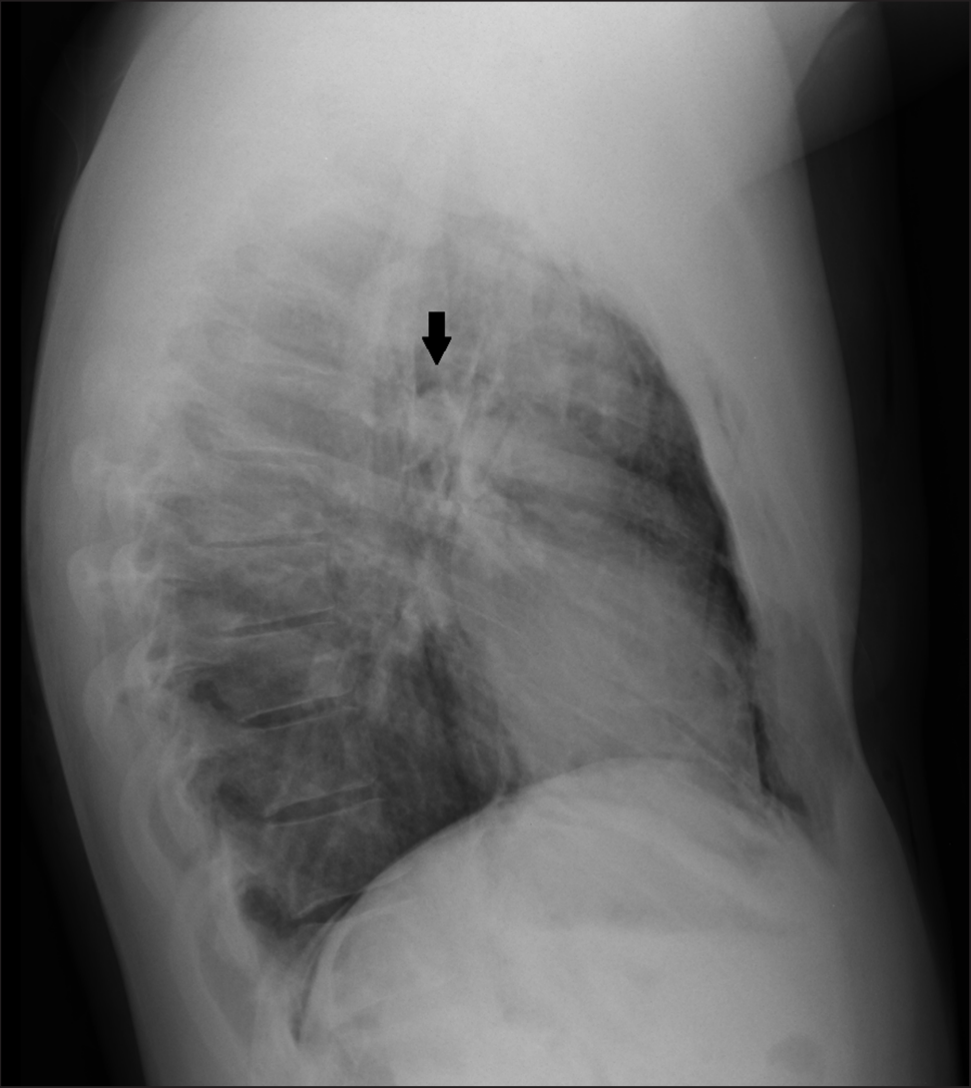
Lateral chest radiograph showing a well-defined lucency surrounding the right pulmonary artery, also known as the ring around the artery sign (solid black arrow).

**Figure C:**
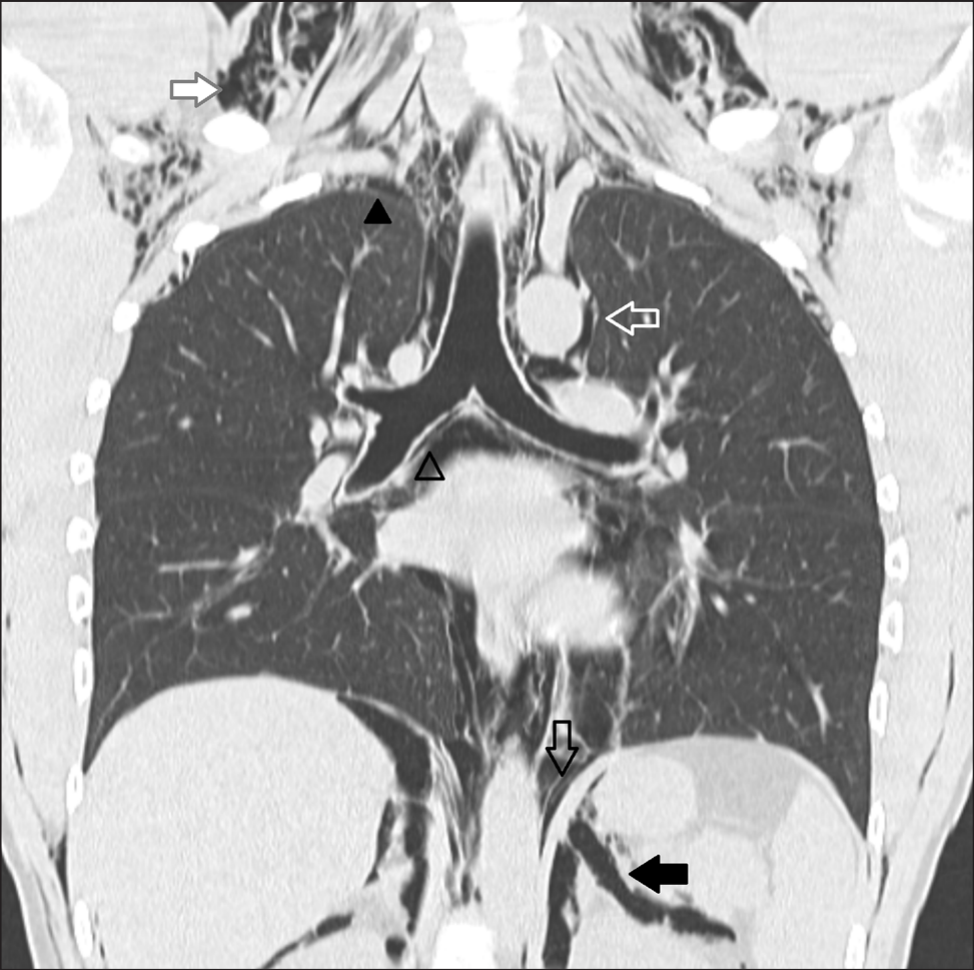
CT of the chest confirming a pneumomediastinum (hollow black arrowhead, hollow black arrow and hollow white arrow) associated with diffuse subcutaneous emphysema (solid white arrow), pneumo(retro)peritoneum (solid black arrow) and pneumothorax (solid black arrowhead).

## Comment

Pneumomediastinum or mediastinal emphysema can occur spontaneously or be secondary to trauma or a pathologic process [1]. Pathophysiologically, pneumomediastinum may result from gas production by microorganisms in acute mediastinitis, perforation of a hollow viscus or the tracheobronchial tree or alveolar rupture due to the existence of a decreasing pressure gradient between the alveoli and the lung interstitium. Spontaneous pneumomediastinum is most commonly associated with inhalational drug use and/or a valsalva-type maneuver [1]. In our case, a spontaneous pneumomediastinum developed after inhaling pepper spray (capsaicin).

The most common symptoms of pneumomediastinum are chest pain, dyspnea and neck pain. Patients with spontaneous pneumomediastinum are usually hemodynamically stable, as was the case in our patient. A nearly pathognomonic sign of pneumomediastinum is Hamman’s crunch, a cracking sound that varies with the heartbeat heard on auscultation of the chest. In our patient, diagnosis of pneumomediastinum was confirmed by anteroposterior and lateral chest radiographs. The radiographic signs that could be observed were subcutaneous emphysema, continuous diaphragm sign, double bronchial wall sign, ring around the artery sign and a tubular artery sign. The continuous diaphragm sign is seen as a radiolucent thin line connecting both hemidiaphragms. The presence of air around the bronchi, the right pulmonary artery and the major aortic branches produces the double bronchial wall sign, ring around the artery sign and the tubular artery sign respectively [1].

A lateral view of the chest is crucial for diagnosis; up to 50% of the cases of pneumomediastinum may remain undiagnosed if only an anteroposterior radiograph is taken. A CT scan of the chest should be reserved for diagnostically uncertain cases.The differential diagnosis of spontaneous pneumomediastinum must consist of pneumothorax, tracheobronchial tree rupture, Boerhaave’s syndrome, pneumopericardium, acute coronary syndrome or pulmonary embolism [1]. CT can already exclude a great bunch of these entities.

Pneumomediastinum can lead to pneumothorax, pneumoperitoneum or pneumo-(retro)peritoneum, which was the case in our patient. Treatment of spontaneous pneumomediastinum is conservative with rest, oxygen therapy, and/or analgesia. The routine use of serial chest radiographs is not indicated and is only recommended in case of a change in the patient’s condition.

## Competing Interests

The authors declare that they have no competing interests.
